# DNA Methylation Levels of the *ELMO* Gene Promoter CpG Islands in Human Glioblastomas

**DOI:** 10.3390/ijms19030679

**Published:** 2018-02-28

**Authors:** Signe Regner Michaelsen, Derya Aslan, Thomas Urup, Hans Skovgaard Poulsen, Kirsten Grønbæk, Helle Broholm, Lasse Sommer Kristensen

**Affiliations:** 1 Department of Radiation Biology, Finsen Center, Rigshospitalet, 2100 Copenhagen, Denmark; Signe.Regner.Michaelsen@regionh.dk (S.R.M.); Thomas.Urup@regionh.dk (T.U.); Hans.Skovgaard.Poulsen@regionh.dk (H.S.P.); 2 Department of Hematology, Rigshospitalet, 2100 Copenhagen, Denmark; deryaaslan87@hotmail.com (D.A.); Kirsten.Groenbaek@regionh.dk (K.G.); 3 Biotech Research and Innovation Centre, BRIC, Copenhagen University, 2200 Copenhagen, Denmark.; 4 Department of Pathology, Center of Diagnostic Investigation, Rigshospitalet, 2100 Copenhagen, Denmark; Helle.Broholm@regionh.dk (H.B.); 5 Department of Molecular Biology and Genetics (MBG), Aarhus University, 8000 Aarhus, Denmark.; 6 Interdisciplinary Nanoscience Center (iNANO), Aarhus University, 8000 Aarhus, Denmark

**Keywords:** glioblastoma, invasion, motility, clinical outcome, DNA methylation, ELMO1, ELMO2, ELMO3

## Abstract

Complete surgical resection of glioblastoma is difficult due to the invasive nature of this primary brain tumor, for which the molecular mechanisms behind remain poorly understood. The three human *ELMO* genes play key roles in cellular motility, and have been linked to metastasis and poor prognosis in other cancer types. The aim of this study was to investigate methylation levels of the *ELMO* genes and their correlation to clinical characteristics and outcome in patients diagnosed with glioblastoma. To measure DNA methylation levels we designed pyrosequencing assays targeting the promoter CpG island of each the *ELMO* genes. These were applied to diagnostic tumor specimens from a well-characterized cohort of 121 patients who received standard treatment consisting of surgery, radiation therapy, plus concomitant and adjuvant chemotherapy. The promoter methylation levels of *ELMO1* and *ELMO2* were generally low, whereas *ELMO3* methylation levels were high, in the tumor biopsies. Thirteen, six, and 18 biopsies were defined as aberrantly methylated for *ELMO1*, *ELMO2,* and *ELMO3*, respectively. There were no significant associations between the methylation status of any of the *ELMO* gene promoter CpG islands and overall survival, progression-free survival, and clinical characteristics of the patients including intracranial tumor location. Therefore, the methylation status of the *ELMO* gene promoter CpG islands is unlikely to have prognostic value in glioblastoma.

## 1. Introduction

Glioblastoma is the most common primary tumor of the central nervous system among adults [1]. Patients typically survive between 12 to 15 months following diagnosis, in spite of extensive treatment with surgery, radiation, and chemotherapy [2,3]. Invasion of tumor cells into the surrounding brain tissue makes complete surgical resection, as well as full radiation of all tumor cells difficult in glioblastoma. This invasive capacity has been linked to fast recurrence of the tumors, which most often manifest within 2 to 3 cm of the resection cavity [4]. The degree of invasiveness does not always correlate with the grade of malignancy, as exemplified by frequent observations of extensive tumor cell infiltration into normal brain tissue in low-grade astrocytomas [5]. The molecular mechanisms behind the aberrant cellular motility of glioma cells remain poorly understood. A better understanding of the factors promoting the invasive nature of glioma cells may lead to identification of prognostic markers and novel targets for treatment.

**Figure 1:**
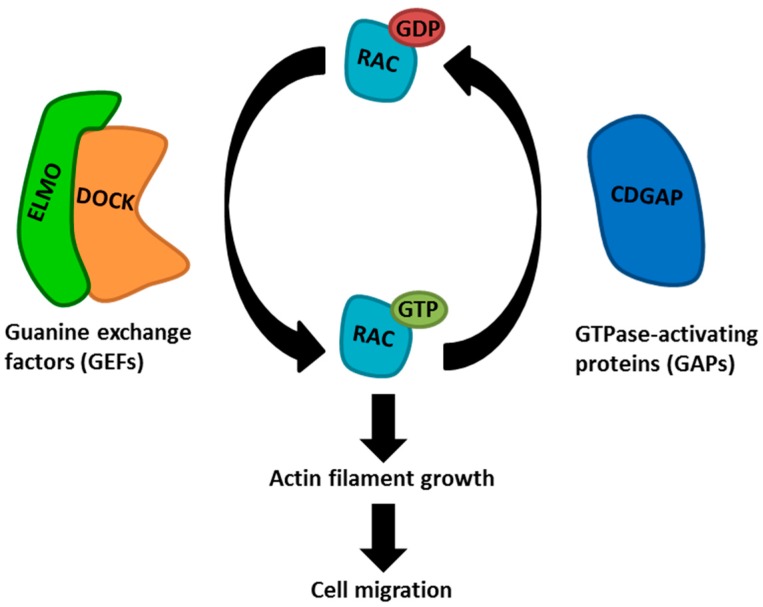
Schematic showing how the ELMO proteins are involved in cell migration. The ELMO proteins function as scaffolds regulating the spatiotemporal localization and activity DOCK guanine exchange factors (GEFs), which promote the active GTP-bound form of RAC proteins. GTPase activating proteins (GAPs) may return RAC to its inactive GDP-bound form. Activated RAC proteins promote actin filament growth, which is required for cell migration.

Recently, it has become apparent that engulfment and cell motility (ELMO) genes play central roles in the dissemination and invasion of cancer cells [6,7,8,9,10,11]. In humans, the ELMO family consists of three proteins, namely ELMO1, ELMO2, and ELMO3, which are involved in cytoskeleton rearrangements during phagocytosis and cellular migration via the activation of Ras-related C3 botulinum toxin substrate (RAC) proteins [12,13,14]. The ELMO proteins lack a catalytic domain but function as scaffolding proteins regulating the spatiotemporal localization and activity of Dedicator Of Cytokinesis (DOCK) guanine exchange factors (GEFs), which are required to promote the active, GTP-bound form of RAC proteins [15]. On the other hand, GTPase activating proteins (GAPs), such as ARHGAP31 (CDGAP) may return RAC to its inactive, GDP-bound state [16,17]. The activation of RAC proteins promotes the growth of actin filaments, which drive cell migration and invasion of cancer cells [10,18,19] (Figure 1). In glioblastoma, ELMO1 has been shown to be specifically abundant in invasive areas of the tumors and to be central for promoting cell migration and invasion via activation of RAC1 [6,20,21]. On the contrary, less is known about the roles of ELMO2 and ELMO3 in glioblastoma.

CpG islands are genomic regions with a high frequency of CpG sites, which are often located near the transcription start site of genes. DNA methylation changes in gene promoter CpG islands are responsible for altering the expression of many different tumor suppressor genes and oncogenes in cancer [22,23,24], including *ELMO1* in gastric cancer [25] and *ELMO3* in lung adenocarcinoma, and associated brain metastases [8]. The detection of specific DNA methylation changes in clinical samples are promising as diagnostic and prognostic biomarkers and for assisting therapeutic decision-making [26,27]. Glioblastomas represent a heterogeneous group of tumors having widely differing genetic- and transcriptional profiles [28,29], but also at the epigenetic level where widespread DNA methylation changes can be observed [30,31]. Specific methylation patterns have been associated with variation in genes that are related to glioblastoma survival and treatment efficacy, including global hypermethylation resulting from isocitrate dehydrogenase (IDH) mutation [31,32] and inverted correlation between promoter methylation and protein expression of *O*^6^-methylguanine-DNA methyltransferase (MGMT) [33]. However, the methylation levels of the *ELMO* gene promoter CpG islands have not previously been reported in this disease.

We hypothesized that methylation levels of the *ELMO* promoter CpG islands in glioblastoma may influence the motility of glioma cells and, therefore, potentially have prognostic value. To investigate this, we developed sensitive and quantitatively accurate assays for DNA methylation detection based on pyrosequencing and studied a well-characterized cohort of 121 patients diagnosed with glioblastoma. Methylation levels of the individual *ELMO* promoter CpG islands were examined for correlation with clinical characteristics of the patients as well as progression-free survival (PFS) and overall survival (OS). In addition, we analyzed publically available methylation- and expression data for the *ELMO* genes.

## 2. Results

### 2.1. Methylation Levels of the ELMO Promoter CpG Islands in Glioblastoma

**Figure 2:**
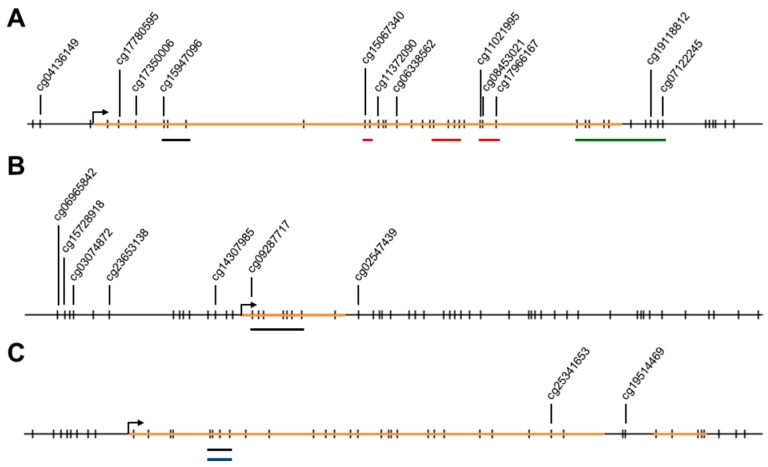
Overview of the CpG sites analyzed for each of the *ELMO* promoters in this and previous studies. The displayed regions each corresponds to 600 bp. Vertical bars represent CpG sites. CpG sites, which have Illumina CpG loci IDs (cg#) are indicated. The orange horizontal lines represent exons. Black bars underline the CpG sites studied here. Red bars underline the CpG sites studied in [25]. The green bar underlines the CpG sites studied in [34]. The blue bar underlines the CpG sites studied in [8]. (**A**) *ELMO1*; (**B**) *ELMO2*; (**C**) *ELMO3*.

The pyrosequencing assays for *ELMO1*, *ELMO2*, and *ELMO3* interrogates three, seven and four individual CpG sites, respectively, within the promoter CpG islands, which include the first exon for all of them (Figure 2).

**Figure 3:**
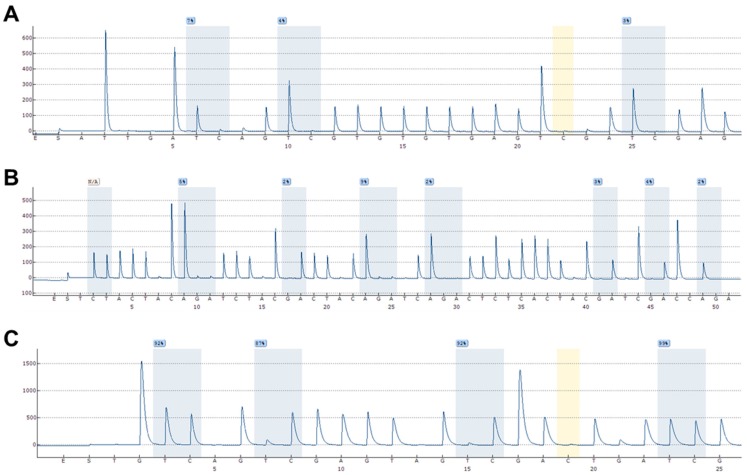
Representative DNA methylation data acquired using pyrosequencing. (**A**) *ELMO1*; (**B**) *ELMO2*; (**C**) *ELMO3*.

Among 121 included glioblastoma patients, successful pyrosequencing results were obtained from 113, 104, and 119 of the patient samples for *ELMO1*, *ELMO2,* and *ELMO3*, respectively. The methylation levels of the three promoter CpG islands for each of the samples can be found in the supplementary materials, while representative results for each of the genes are shown in Figure 3. 

**Figure 4:**
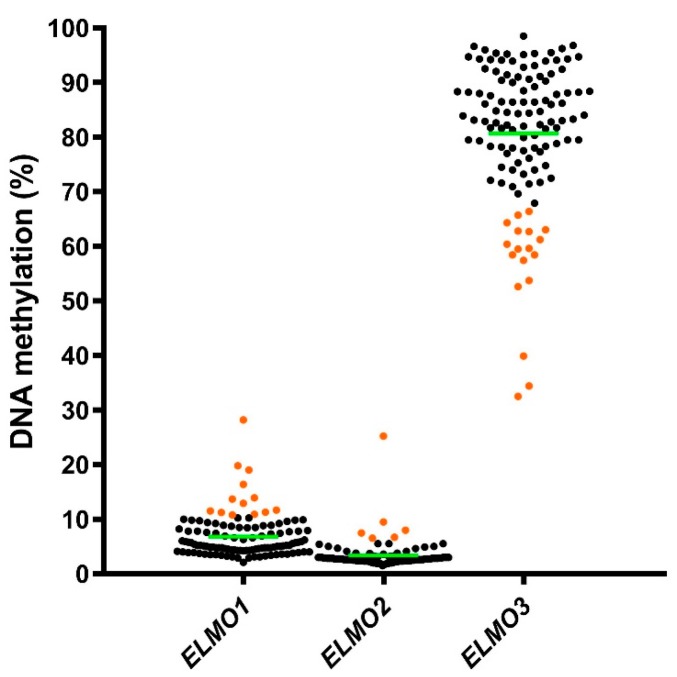
DNA methylation levels in the glioblastoma samples. Hypermethylated cases for *ELMO1* and *ELMO2* and hypomethylated cases for *ELMO3* are indicated in orange color. The green bars indicate mean methylation levels for each gene promoter CpG island.

The mean *ELMO1*, *ELMO2,* and *ELMO3* methylation levels in the tumor biopsies were 6.8% (SD 3.9), 3.3% (SD 2.6), and 80.6% (SD 13.0), respectively. Thirteen and six samples had methylation levels greater than one SD above the cohort mean for *ELMO1* and *ELMO2*, respectively, and were defined as hypermethylated. Eighteen samples had methylation levels greater than one SD below the cohort mean for *ELMO3* and were defined as hypomethylated (Figure 4).

**Figure 5:**
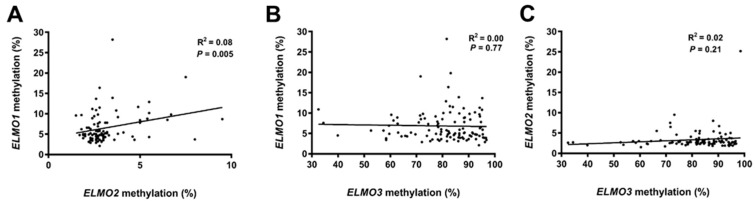
Correlations between the methylation levels of the individual *ELMO* gene promoter CpG islands. (**A**) *ELMO1* vs. *ELMO2*; (**B**) *ELMO1* vs. *ELMO3*; (**C**) *ELMO2* vs. *ELMO3*.

We further investigated if patients with an aberrantly methylated sample for one of the *ELMO* gene promoter CpG islands were more likely to have aberrant methylation for one of the other CpG islands. This was not the case as only a weak correlation was found between *ELMO1*- and *ELMO2* methylation levels. On the other hand, the methylation levels of none of these genes were significantly correlated with *ELMO3* methylation levels (Figure 5).

### 2.2. Methylation Levels of the ELMO Gene Promoters in Normal Tissues

Methylation levels of the *ELMO* gene promoter CpG islands in normal tissues were investigated by consulting publically available data sets using the R2: Genomics Analysis and Visualization Platform (http://r2.amc.nl). The *ELMO1* (cg15947096) and *ELMO2* (cg09287717) promoter CpG islands were found to be methylated at low levels (average (avg.) 11% and avg. 4%, respectively) in all ten different human tissues that were analyzed by Slieker and co-workers [35]. Likewise, the *ELMO2* promoter CpG island (cg09287717) was found to be methylated at low levels (avg. 2%) in all 17 different human tissues, including medulla oblongata and ischiatic nerve, as analyzed by Lokk and co-workers [36]. None of the CpG sites that we analyzed in the *ELMO3* promoter CpG Island using pyrosequencing were included in these data sets. However, nearby CpG sites (cg25341653 and cg19514469) were methylated at relatively high levels (avg. 63% and avg. 52%, respectively) in all of the tissues analyzed by Slieker and co-workers [35], and in all tissues analyzed by Lokk and co-workers (avg. 65% and avg. 48%, respectively) [36]. Altogether, these analyses indicate that low level methylation of the *ELMO1* and *ELMO2* promoter CpG islands, and high level methylation of the *ELMO3* promoter CpG Island are normal, whereas hypermethylation of *ELMO1* and *ELMO2* and hypomethylation of *ELMO3* are likely to be associated with a malignant phenotype. In support of this, using the R2: Genomics Analysis and Visualization Platform, we also found that *ELMO1* and *ELMO2* are expressed at high levels, whereas *ELMO3* is expressed at low levels, in normal brain tissues analyzed by Berchtold and co-workers [37].

### 2.3. DNA Methylation Status of the ELMO Promoter CpG Islands According to Patient Characteristics

The baseline clinical characteristics of the glioblastoma patients as a function of the methylation status of the respective *ELMO* promoter CpG islands are shown in Table 1. There was no significant association between the methylation status of any of the *ELMO* promoter CpG islands and any of the examined clinical characteristics, including age, gender, WHO performance status, diagnosis, use of corticosteroids at treatment start, multifocal disease, tumor brain location, site of relapse tumor, or *MGMT* promoter methylation. However, tendencies were observed that patients with an *ELMO1* hypermethylated tumor were unmethylated at the *MGMT* promoter (*p* = 0.053), and likewise that patients with an *ELMO3* hypomethylated tumor were unmethylated at the *MGMT* promoter (*p* = 0.137).

### 2.4. Survival Analyses According to DNA Methylation Status of the ELMO Genes

Univariate analysis of *ELMO* CpG island methylation status with survival endpoints found no statistically significant differences in OS or PFS of the patients according to the methylation status of any of the *ELMO* promoter CpG islands (Table 2). However, a tendency was observed for a shorter PFS in patients with an *ELMO3* hypomethylated tumor (Hazard ratio; 1.48, 95% confidence interval; 0.89–2.47, *p* = 0.129).

## 3. Discussion

In glioblastoma, the poor outcome of patients has been linked to the invasive nature of the cancer. The association of the *ELMO* genes to cancer cell migration primed us to profile the methylation levels of all three human *ELMO* genes in a cohort of 121 glioblastoma patients, and examine if aberrant methylation of the *ELMO* genes is correlated to patient clinical characteristics and survival. The ability to assess methylation levels quantitatively is crucial for establishing specific assays for clinical use [38,39,40]. Therefore, we used pyrosequencing, which is a quantitatively accurate method [41] that is already in clinical use for assessment of *MGMT* methylation levels in glioblastoma. We found that the methylation levels of *ELMO1* and *ELMO2* were generally low, whereas *ELMO3* methylation levels were high, in the glioblastoma samples. However, some patient samples were hypermethylated for *ELMO1* and *ELMO2*, and some patient samples were hypomethylated for *ELMO3* (Figure 4). This corresponds well with previous findings from other types of cancer, where promoter hyper- and hypomethylation have been observed in a subset of cases for *ELMO1* and *ELMO3*, respectively [8,25,34]. On the contrary, this study is, to our knowledge, the first to describe methylation of the *ELMO2* promoter in cancer. In addition, we found that *ELMO1* and *ELMO2* are methylated at very low levels across a large number of different non-cancerous human tissues, whereas as *ELMO3* is normally methylated at high levels. Therefore, it is likely that the observed hypermethylation in a subset of cases for *ELMO1* and *ELMO2*, and hypomethylation in a subset of cases for *ELMO3*, represent events that are associated with the malignant phenotype. We have previously observed that the methylation level of the *ELMO3* promoter CpG Island is inversely correlated with expression of the gene [8], however, such potential correlations have not been investigated for *ELMO1* and *ELMO2*.

To assess whether the methylation levels of the *ELMO* genes have an impact on outcome in glioblastoma we used cut-offs to separate the patients into two groups for each of the genes. These cut-offs were defined as one standard deviation above the cohort mean for *ELMO1* and *ELMO2* and as one standard deviation below the cohort mean for *ELMO3* (Figure 4). We found that this was more appropriate for the data of this study in comparison to the use of medians as cut-offs, as this implied that the groups of high- and low methylation contained samples of very similar methylation levels. In addition, we have previously successfully used one standard deviation below the cohort mean as cut-off in a study of LINE-1 methylation levels in diffuse large B-cell lymphoma [40], supporting use of this cut-off. Using these pre-defined cut-offs, we did not observe any statistically significant correlations between *ELMO* methylation status and neither patient- and tumor characteristics nor survival of the patients. This included the presence of multifocal disease and a site for the recurrent tumor distant from the primary site. This argues against the ELMO variants being a central driver of glioblastoma intracranial spread. Still, there was a tendency for patients with a hypomethylated *ELMO3* promoter to have shorter PFS. High expression of *ELMO3* have been linked with poor prognosis in lung cancer [42], head and neck squamous cell carcinoma [43]**,** and laryngeal cancer [44], and we have previously shown that methylation levels of the *ELMO3* promoter is inversely correlated with expression of the gene [8]. Thus, this could support that our data reflect a worse therapeutic response for patients having a tumor with a hypomethylated *ELMO3* promoter. However, we also noted a tendency that hypomethylation of *ELMO3* co-existed with an unmethylated *MGMT* promoter. Since glioblastoma patients receiving Stupp’s regimen having an unmethylated *MGMT* promoter present shorter PFS and OS [2,33]**,** this may also explain the shorter PFS of patients with *ELMO3* hypomethylation.

In conclusion, we have characterized the methylation levels of several individual CpG sites in the three human *ELMO* gene promoter CpG islands in a large well-characterized cohort of glioblastoma patients. Aberrant methylation levels were only present in a small subset of patients and were not associated with OS, PFS, or clinical characteristics of the patients. Therefore, the methylation status of the *ELMO* genes is unlikely to have prognostic value in glioblastoma. However, we cannot exclude that different results may be obtained if other CpG sites within the *ELMO* gene promoters are analyzed, and additional cohorts should be analyzed to firmly establish that the *ELMO* genes do not play a role in the pathogenesis of glioblastoma.

## 4. Materials and Methods

### 4.1. Patient Samples

This retrospective study examined material from 121 patients diagnosed with glioblastoma (World Health Organization (WHO) grade IV), according to the WHO 2000/2007 guidelines from 2005 to 2010. In addition to a diagnosis with glioblastoma, patient inclusion criteria were that tumor DNA was available and that patients had received primary standard glioblastoma therapy according to the Stupp regimen (concomitant radiation and temozolomide therapy followed by up to six courses of adjuvant temozolomide therapy) at Rigshospitalet, Denmark. This patient material has previously been used in a study investigating *MGMT* methylation patterns in glioblastoma [33] and the *MGMT* data analyzed here is from this study. 

For most patients, primary operation consisted of either partial resection (64 patients) or gross total resection (53 patients), while three patients were biopsied only (data missing, *n* = 1). Median number of adjuvant courses of temozolomide therapy was four, and best clinical response on Stupp treatment was partial response for 16 patients, stable disease for 53 patients, and progressive disease for 47 patients (data missing, *n* = 5). After progression on primary therapy, 51 of the 121 patients received reoperation (data missing, *n* = 8), while 59 of the 121 patients received various types of palliative therapy (data missing, *n* = 9), of which 53 received avastin combined with irinotecan. The median duration of observation from the day patients first received radiation/temozolomide therapy to the project cutoff day (2 February 2017) was 112 months (range, 73–142 months). At this time point, three patients were still alive, of which two had not progressed on Stupp treatment. More patient characteristics are shown in Table 1, while detailed descriptions of the treatments and patient evaluations have been described elsewhere [45].

### 4.2. Ethics Statement

This study was performed according to the Declaration of Helsinki and Danish legislation. Permissions were given from the Danish Data Protection Agency (2015-41-4118, 01. 09. 2015) and the ethical committee for the Capital Region of Denmark (H-C-2008-095, 10. 10. 2008).

### 4.3. DNA Purification and Sodium Bisulfite Treatment

DNA was obtained from fresh frozen tumor tissue obtained from the primary diagnostic glioblastoma surgery before exposure to radiation or chemotherapy treatment. Approximately 50 mg homogenized tissue was used for extraction by employing standard Proteinase K-Phenol/Chloroform extraction and sodium bisulfite conversion as described [33].

### 4.4. DNA Methylation Analyses Using Pyrosequencing

**Figure 6:**
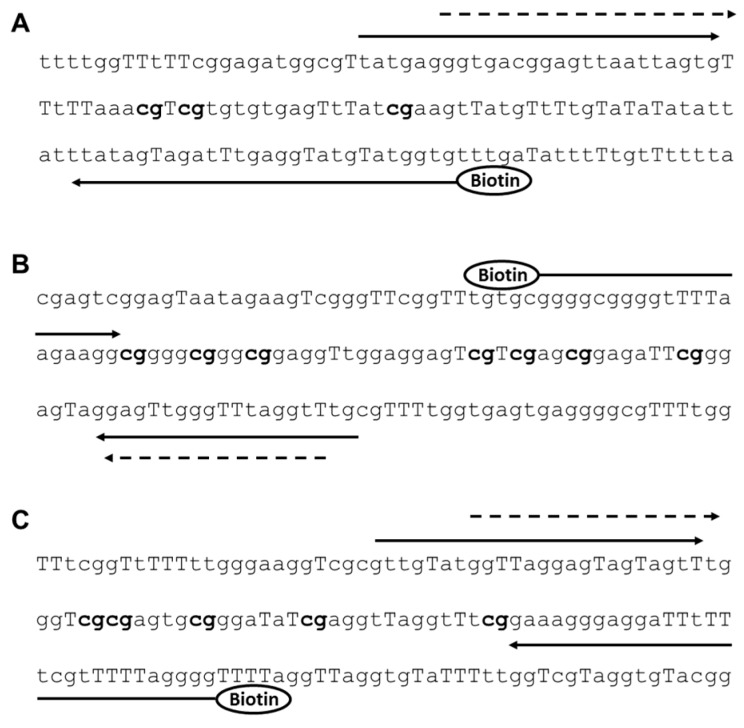
Primer design for each of the pyrosequencing assays. PCR Primes are denoted as solid arrows and sequencing primers as dashed arrows. Uppercase T denotes cytosines, which have been converted to uracil during the sodium bisulfite treatment. The CpG sites analyzed are indicated in bold. (**A**) *ELMO1*; (**B**) *ELMO2*; (**C**) *ELMO3*.

Pyrosequencing is a quantitatively accurate method for the analyses of DNA methylation at single nucleotide resolution [39,46,47]. We designed and optimized novel pyrosequencing assays for the promoter regions of *ELMO1*, *ELMO2* and *ELMO3*, respectively, based on methylation independent PCR (MIP) primers [48] (Figure 6). The PCR and sequencing primers were designed using the PyroMark Assay Design 2.0 software (Qiagen, Hilden, Germany) and the sequences are listed in Table 3. PCR was performed on the Gene PCR System 9700 (Applied Biosystems, Foster City, CA, USA). The PyroMark PCR Master Mix (Qiagen) was used, according to the manufactures’ instructions, with primer concentrations of 200 nM and 1 μL bisulfite converted DNA was used as template. The cycling protocol started with 1 cycle of 95 °C for 10 min, followed by 45 cycles of 95 °C for 5 s, 58 °C for 10 s, 72 °C for 10 s. Samples were sequenced on the PyroMark Q24 (Qiagen) using the PyroMark Gold Q24 reagents (Qiagen), according to the manufactures’ instructions. Methylated DNA (Chemicon, Millipore, Billerica, MA, USA), unmethylated DNA (Qiagen), 50% methylated DNA and no template controls (NTCs) were included in all of the experiments. For each gene, the mean methylation levels of the analyzed CpG sites were used in subsequent analyses. Hyper- and hypomethylation was defined as a methylation level above or below the cohort mean methylation level plus or minus one standard deviation, respectively. The cutoffs were 10.7, 5.9, and 67.6% for *ELMO1*, *ELMO2,* and *ELMO3*, respectively. Data were only included in subsequent analyses if there was no warnings associated with the analyses of methylation levels using the pyrosequencing software with default settings.

### 4.5. Data Availability

The methylation levels for each of the *ELMO* gene promoter CpG islands for each of the samples can be found in the supplementary materials.

### 4.6. Statistical Analyses

Statistical analyses were performed in SPSS 22.0 for Windows (SPSS Inc., IBM Corp., Armonk, NY, USA) and in Prism 6 (GraphPad software, San Diego, CA, USA). Goodness-of-fit linear regression was used to evaluate the possible relations between methylation levels of the individual *ELMO* genes and by employing an F test to evaluate if the slopes were significantly different from zero. Comparisons of clinical characteristics between patients with hypermethylation/hypomethylation and normal methylation levels for the *ELMO* gene promoter CpG islands were done using the Fisher’s exact test and the Mann-Whitney U test. Survival probabilities (PFS and OS) were estimated using the Kaplan-Meier method, while the Cox proportional hazards model was used for univariate analyses of *ELMO* methylation level and PFS and OS, respectively, for which the results are presented as hazard ratios (HR) with 95% confidence interval (CI). Any differences were considered to be statistically significant when the *p* value was <0.05.

## Figures and Tables

**Table 1:** DNA Methylation Status of the *ELMO* Genes According to Patient Characteristics.

Clinical Variable	All (*n* = 121)	*ELMO1* Hypermet (*n* = 13)	*ELMO1* Normal Met (*n* = 99)	*p*-Value	*ELMO2* Hypermet (*n* = 6)	*ELMO2* Normal Met (*n* = 97)	*p*-Value	*ELMO3* Hypomet (*n* = 18)	*ELMO3* Normal Met (*n* = 101)	*p*-Value
Age (years), median (range)	59.0 (23–74)	58.0 (40–67)	59.0 (23–74)	0.898	56.0 (46–71)	60 (23–74)	0.728	60.0 (23–74)	58.0 (31–72)	0.602
Gender, *n* (%)										
Female	39 (32.2)	4 (30.8)	34 (34.3)	1.000	3 (50.0)	31 (32.0)	0.394	8 (44.4)	30 (29.7)	0.273
Male	82 (67.8)	9 (69.2)	65 (65.7)		3 (50.0)	66 (68.0)		10 (55.6)	71 (70.3)	
WHO performance status, *n* (%)										
0	69 (57.0)	8 (61.5)	58 (61.7)	1.000	4 (66.7)	57 (62.0)	1.000	9 (52.9)	59 (61.5)	0.594
1–2	46 (38.1)	5 (38.5)	36 (38.3)		2 (33.3)	35 (38.0)		8 (47.1)	37 (38.5)	
Missing	6 (4.9)	0	5		0	5		1	5	
Diagnosis, *n* (%)										
Primary Glioblastoma	116 (95.9)	13 (100.0)	94 (94.9)	1.000	6 (100.0)	92 (94.8)	1.000	18 (100.0)	96 (95.0)	1.000
Secondary Glioblastoma	5 (4.1)	0 (0.0)	5 (5.1)		0 (0.0)	5 (5.2)		0	5 (5.0)	
Corticosteroid use, *n* (%)										
Yes	86 (71.1)	10 (76.9)	71 (72.4)	1.000	5 (100.0)	66 (68.8)	0.318	12 (66.7)	73 (73.7)	0.570
No	33 (27.3)	3 (23.1)	27 (27.6)		0 (0.0)	30 (31.3)		6 (33.3)	26 (26.3)	
Missing	2 (1.7)	0	1		1	1		0	2	
Multifocal Disease, *n* (%)										
Yes	8 (6.6)	0 (0.0)	7 (7.1)	1.000	1 (16.7)	7 (7.2)	0.392	2 (11.1)	5 (5.0)	0.286
No	113 (93.4)	13 (100.0)	92 (92.9)		5 (83.3)	90 (92.8)		16 (88.9)	96 (95.0)	
Tumor brain location, *n* (%)										
Frontal	26 (21.5)	1 (7.7)	23 (23.2)	0.292	3 (50.0)	22 (22.7)	0.152	5 (27.8)	19 (18.8)	0.358
Other	95 (78.5)	12 (92.3)	76 (76.8)		3 (50.0)	75 (77.3)		13 (72.2)	82 (81.2)	
Site of relapse tumor, *n* (%)										
Local in primary site	70 (57.9)	8 (80.0)	58 (84.1)	0.666	4 (100.0)	54 (83.1)	1.000	13 (86.7)	57 (85.1)	1.000
Distant from primary site	13 (10.7)	2 (20.0)	11 (15.9)		0 (0.0)	11 (16.9)		2 (13.3)	10 (14.9)	
Missing	38 (31.4)	3	30		2	32		3	34	
*MGMT* promoter methylation, *n* (%)										
Yes	38 (31.4)	1 (8.3)	35 (38.5)	0.053	2 (40.0)	31 (34.8)	1.000	2 (14.3)	34 (35.8)	0.137
No	73 (60.3)	11 (91.7)	56 (61.5)		3 (60.0)	58 (65.2)		12 (85.7)	61 (64.2)	
Missing	10 (8.3)	1	8		1	8		4	6	

Statistical tests: Mann-Whitney U test (Age); Fisher’s exact test (Gender, WHO performance status, Diagnosis, Corticosteroid use at start treatment, Multifocal disease, Tumor brain location, Site of relapse tumor, *MGMT* promoter methylation). Abbreviations: met (methylation).

**Table 2:** Survival analyses according to DNA methylation status of the *ELMO* genes.

Clinical Endpoint	All (*n* = 121)	*ELMO1* Hypermet (*n* = 13)	*ELMO1* Normal Met (*n* = 99)	HR (95% CI) *p*-Value	*ELMO2* Hypermet (*n* = 6)	*ELMO2* Normal Met (*n* = 97)	HR (95% CI) *p*-Value	*ELMO3* Hypomet (*n* = 18)	*ELMO3* Normal Met (*n* = 101)	HR (95% CI) *p*-Value
OS (mo), median (range)	14.0 (1–131)	14.0 (4–31)	14.0 (1–131)	1.12 (0.62–2.01) *p* = 0.71	10.0 (1–27)	14.0 (1–131)	1.32 (0.57–3.03) *p* = 0.530	12.0 (7–43)	14.0 (1–131)	1.16 (0.70–1.92) *p* = 0.567
PFS (mo), median (range)	6.0 (0–131)	7 (2–21)	7 (0–131)	1.03 (0.58–1.85) *p* = 0.91	9.0 (1–16)	6.0 (0–131)	1.15 (0.50–2.64) *p* = 0.75	4.0 (3–30)	7.0 (0–131)	1.48 (0.89–2.47) *p* = 0.129

Statistical tests: Kaplan-Meier method for estimation of OS and PFS using the Cox proportional hazards model. Abbreviations: met (methylation); OS (overall survival); PFS (progression-free survival); HR (Hazard Ratio); mo (months).

**Table 3:** Primer sequences and details of the pyrosequencing assays.

Gene Name	Primers (5′→3′)	Amplicon Size (bp)
*ELMO1*	Forward primer: TATGAGGGTGAAGGAGTTAATTAGTG Reverse primer: Biotin-CACCATACATACCTCAAATCTACTATAA Sequencing primer: AGGGTGAAGGAGTTAATTAGT	107
*ELMO2*	Forward primer: biotin-GGGGAGGGGTTTTAAGAAGG Reverse primer: CAAACCTAAACCCAACTCC Sequencing primer: AACCTAAACCCAACTC	87
*ELMO3*	Forward primer: GTTGTATGGTTAGGAGTAGTAGTT Reverse primer: Biotin-CCCCTAAAAACCAAAAAATCCTCCCTTTC Sequencing primer: GGTTAGGAGTAGTAGTTT	89

## Supplementary Materials

Supplementary materials can be found at http://www.mdpi.com/1422-0067/19/3/679/s1.
